# Excitation of Mechanical Resonances in the Stationary Ring of a Mechanical Seal by a Continuously Operated Electromagnetic Acoustic Transducer

**DOI:** 10.3390/s23021015

**Published:** 2023-01-16

**Authors:** Alexander Siegl, Stefan Leithner, Bernhard Schweighofer, Hannes Wegleiter

**Affiliations:** 1 Institute of Electrical Measurement and Sensor Systems, Graz University of Technology, 8010 Graz, Austria; 2 Institute of Hydraulic Fluidmachinery, Graz University of Technology, 8010 Graz, Austria

**Keywords:** electromagnetic acoustic transducer, EMAT design, EMAT simulation, bulk-wave EMAT, structural analysis, vibration measurement, mechanical seals

## Abstract

Acoustic/ultrasonic testing is now a common method in the field of nondestructive testing for detecting material defects or monitoring ongoing mechanical changes in a structure during operation. In many applications, piezoelectric transducers are used to generate mechanical waves inside the specimen. Their actual operating frequency is highly dependent on the dimensions of the transducer. Larger dimensions of the piezoelectric transducer allow for a lower operating frequency. However, these dimensions limit the use of piezoelectric transducers in certain applications where the size of the transducer is restricted due to limited installation space and when low-frequency excitation is required. One application that places these requirements on the transducer is the monitoring of mechanical seals. Here, the transducer must be mounted on the stationary ring of the seal. In this paper, a continuously operated electromagnetic acoustic transducer (EMAT) is presented as an alternative to piezoelectric transducers as a transmitter. The advantage of a EMAT is that it meets the requirements of limited sensor size (sensor area < 10 × 6 mm) and can excite mechanical waves with frequencies below 10 kHz. A structural analysis of the stationary ring shows that the first two mechanical resonances occur around 4 and 5.5 kHz. An experimental study meterologically demonstrates the ability of the EMAT to excite these first two mechanical resonances of the ring. A comparative simulation agrees well with the measurement.

## 1. Introduction

**Figure 1:**
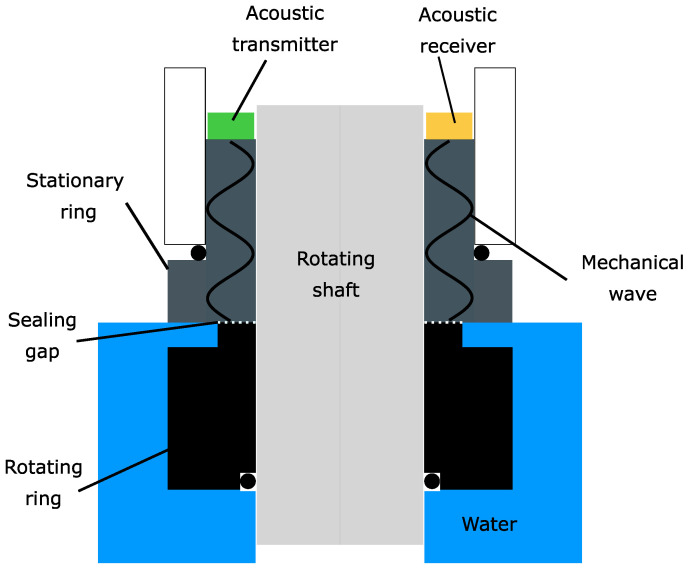
Schematic representation of a mechanical seal. The seal consists of a stationary ring and a rotating ring attached to the rotating shaft. The seal faces form the sealing gap. For mechanical monitoring purposes, an acoustic transmitter and a receiver are attached on the back side of the stationary ring. The acoustic transmitter incorporates a mechanical wave inside the stationary ring. This wave then propagates in the stationary ring and is measured by the acoustic receiver.

Mechanical seals are widely used today when housing needs to be sealed against a rotating shaft [1]. A typical application is the sealing of a centrifugal pump [2,3]. A mechanical seal consists of a stationary ring and a rotating ring and is mounted on the rotating shaft. The faces of each ring form the sealing gap. The process fluid inside the housing and the rotation cause a lubricating film to form in the sealing gap. This lubricating film forms the actual sealing point of the mechanical seal. During operation, the thickness of the lubricating film decreases, and increased wear of the seal occurs due to increased solid contact in the sealing gap [4]. The condition of this contact situation in the sealing gap is decisive for the remaining lifetime of the seal. To prevent an unscheduled stop of the machine due to a seal failure [5], multiple monitoring approaches for seals have been reported [6]. One possible monitoring approach is to measure the stationary ring temperature [7,8,9]. Another approach is to measure the electrical resistance through the seal gap to obtain information about the contact situation [10]. Other monitoring approaches measure the acoustic emissions generated by the sliding rings [11,12,13]. However, a drawback of this approach is that other acoustic sources interfere with the measurement result. To overcome this problem, acoustic/ultrasonic monitoring methods in which a transmitter actively sends a mechanical wave into the ring have been extensively studied in recent years [14,15].Figure 1 shows a schematic image of a mechanical seal with attached instrumentation for acoustic/ultrasonic monitoring using an active acoustic transmitter.

**Figure 2:**
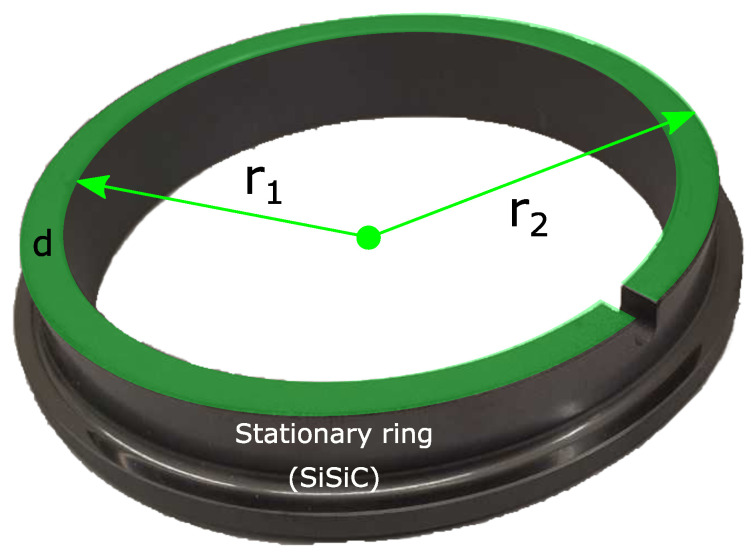
Schematic of the stationary ring used in this study. A transmitter has to be placed on top of the ring and inside the green area. The thickness *d* is given through the inner radius $r_{1}$ and the outer radius $r_{2}$. The radial dimensions of the sensors must no be larger than the thickness *d*.

The instrumentation consists of an acoustic transmitter and receiver. The transmitter generates a mechanical wave inside the stationary ring. The wave propagates in the ring and is measured by the receiver. In this case, the transmitter and receiver must be mounted on the back of the stationary ring to allow possible use in the field. A closer view of a stationary ring is given in Figure 2. The mounting area for the transmitter is highlighted in green. It is a circular ring area. Its thickness is determined through the inner radius $r_{1}=50.5\;mm$ and the outer radius $r_{2}=56.5\;mm$. Hence, the resulting thickness equals d=6 mm. The transmitter mounted on the ring must not exceed this thickness; otherwise, the ring equipped with the instrumentation cannot be installed in a possible test bench or used in a practical application.

In a related study, the transmitter and receiver used for acoustic/ultrasonic monitoring approaches were piezoelectric transducers (PZTs) [16,17,18,19,20,21]. PZTs are used to incorporate pulsed mechanical wave packets into the ring. Depending on the contact situation [22,23] between the rings, the amplitude of the measured mechanical pulse is affected [24,25,26].

However, due to application-specific limitations, PZTs can often reach their limits. These situations include, for example, hot surfaces that exceed the maximum operating temperature of the piezo material and the adhesive used to mount the PZT [27,28]. Another limitation often arises due to the available installation space. The mechanical pulses emitted by the PZT strongly depend on the resonance frequency [29,30]. In order to obtain high mechanical efficiency, the transducer is typically excited in the vicinity of its resonance frequency. The resonant frequency of a PZT depends, among other things, on its size. For example, a commercially available piezoelectric disc with a diameter of 5 mm that fits on the ring has to be operated at its radial resonant frequency, usually around 400 kHz [31]. In general, the lower the required resonant frequency, the larger the transducer is built. This dependence between the size of the transducer and its resonant frequency, as well as the temperature limitation already mentioned, limit the universal use of PZTs for monitoring mechanical seals. To overcome these limitations, we propose the use of an electromagnetic acoustic transducer (EMAT) as the transmitter [32,33].

An EMAT provides an alternative way to generate mechanical waves in structures [34,35,36,37,38]. In this paper, we show that an EMAT can solve the listed problems that a PZT has in monitoring a mechanical seal. In particular, the limitations in the choice of operating frequency of a PZT due to its size dependence can be overcome by an EMAT. This advantage of choosing the operating frequency independent of the transducer size further opens up a monitoring approach where we can excite the fundamental mechanical resonances of the stationary ring. As we show in this paper, the first fundamental resonances already occur at frequencies below 10 kHz. The resonant vibrations at these frequencies make it easier to understand the actual direction of oscillation of the ring. In addition, nicely spatially separated oscillating antinodes form, allowing a defined positioning of the instrumentation compared with an excitation, e.g., at 400 kHz.

The proposed EMAT in this study acts as a transmitter that spatially fits on the stationary ring and is capable of exciting the first mechanical resonances of the ring, despite its compact design. In this regard, we also present a new method of operating the EMAT. The state of the art in EMAT operation is the use of a high-current ultrasonic pulse to generate a single mechanical pulse that propagates inside the sample [39,40]. In our study, however, we found that it is possible to drive the EMAT with a continuous sinusoidal excitation current to excite the mechanical resonances of the ring. To the best of our knowledge, this type of EMAT operation has never been reported.

The focus of this study was on EMAT design and the specific excitation and understanding of the fundamental mechanical resonances of the stationary ring. The actual use of these mechanical resonances to investigate a possible interaction between the stationary ring and the rotating ring is considered a separate research topic that is currently in progress.

The paper is structured as follows: In the first section, the general working principle of the EMAT is briefly described and the actual EMAT design for the stationary ring is presented. In the next section, three simulation studies are presented. In these simulation studies, the eigenfrequencies and the corresponding mode shapes of the stationary ring were elaborated and the interaction between the EMAT and the ring was simulated. Within this section, the position of the EMAT and the receiver is described. In the third section, an experimental study is presented in which the EMAT excites the mechanical resonances of the ring.

## 2. EMAT Working Principle and Design for the Stationary Ring

As mentioned in the Introduction, an EMAT provides an alternative way to excite or detect mechanical waves inside a solid material. It can be built in a compact way and, depending on its design, an EMAT can be capable of exciting mechanical waves at frequencies lower than 10 kHz. In this study, an EMAT was used as transmitter to continuously generate mechanical waves inside the stationary ring. In general, there are three possibilities for the EMAT to incorporate mechanical excitation in an electrically conductive or ferromagnetic material.

The first possibility is the generation of Lorentz forces based on the electrical conductivity of the material. The second and third possibilities are referred to as magnetization and magnetostriction due to the ferromagnetic properties of the material. In this study, the specimen under test was the stationary ring of a mechanical seal. The ring was made of reaction-bonded silicon (siliconized silicon carbide SiSiC) [41]. This material is an electrical insulator and nonferromagnetic. So, none of the three possibilities to incorporate mechanical excitation function inside a SiSiC. However, Hsu et al. [42] already showed that an EMAT can be used for nonconductive and nonferromagnetic specimens by applying a coupling medium between the EMAT and the specimen. Hsu et al. used aluminum adhesive tape as the coupling medium. Aluminum is an electrically conductive material, so Lorentz forces are generated inside the tape. These Lorentz forces then generate mechanical waves inside the aluminum and propagate from the aluminum tape into the specimen. In order to generate Lorentz forces, an EMAT consists of three components:

1. A coil (often printed on a circuit board);
2. A static magnetic field source;
3. A conductive material (e.g., aluminium as the coupling medium).

In a first step, a time-varying current is driven through the coil. This current generates a time-varying magnetic field that induces eddy currents $J_{e}$ inside the aluminum. In a second step, the induced eddy currents then interact with a static magnetic field $B_{0}$ provided by the static magnetic field source. The Lorentz forces $F_{L}$ are generated according to Equation (1).

(1) $$F_{L}=J_{e}\times B_{0}$$

Depending on the EMAT design, the direction of the Lorentz forces and the resulting mechanical waves propagating in the specimen are different. In the following section, we focus on a particular EMAT design with its constraints in terms of spatial installation space on the stationary ring.

### EMAT Design for the Stationary Ring

**Figure 3:**
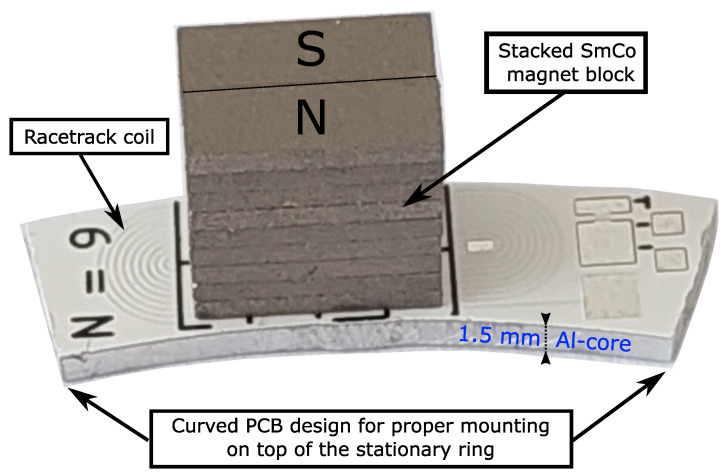
EMAT design for the stationary ring. The curved PCB fits the radius of the stationary ring for edgeless mounting. The racetrack coil consists of 9 turns and a block magnet built of 20 single SmCo magnets is centered with respect to the racetrack coil and glued on top of the PCB. The core of the PCB is formed by a 1.5 mm aluminum layer. Within this layer, Lorentz forces are generated, and the mechanical waves are guided into the stationary ring.

As mentioned in the Introduction, the challenge of this particular measurement task is the limited mounting space for possible sensors on the ring. In particular, for the EMAT, this means the maximum width of the printed circuit board (PCB) of the EMAT is spatially predefined. Therefore, the coil manufactured on the PCB is also limited in the number of turns. Figure 3 shows the EMAT for the stationary ring. The design used for the EMAT is referred to as bulk-wave EMAT [32]. A bulk-wave EMAT consists of an elongated spiral coil (racetrack coil) manufactured on a PCB and a block magnet on top of the racetrack coil. The racetrack coil consists of N=9 turns. In the middle of the PCB, the turns of the coil run parallel in order to generate an enclosed area of induced eddy currents. The shape of the PCB was adjusted in order to fit the inner and outer radii of the ring. On top of the coil, 10 single samarium-cobalt (SmCo) magnets were stacked on each side of the coil and formed the permanent magnet. This magnet block was glued onto the PCB and provided the static magnetic field for the Lorentz force generation.

The novelty of the proposed EMAT is the material used for the PCB core. Typically, the core material of the PCB is an FR-4 material. However, because an aluminum coupling layer is required to generate Lorentz forces and guide the mechanical waves into the ring, an aluminum core with a thickness of 1.5 mm was chosen as the core material for the circuit board. By using the aluminum core, the EMAT can be considered a stand-alone transmitter, similar to a piezoelectric transmitter. However, the advantage of the EMAT is the free choice of excitation frequency, while being compactly built.

This EMAT was later used to mechanically excite the resonances of the stationary ring. However, first, we had to find the frequencies where these resonances occur. A modal analysis of the stationary ring provided the natural eigenfrequencies at which the ring mechanically resonates.

## 3. Modal Analysis of the Stationary Ring and EMAT Simulation

The objective of this study was to mechanically excite certain resonances of the ring using the EMAT. This section is divided into three subsections in which various simulation studies are described.

- First, we had to identify the frequencies where the resonances of the ring occur. This was achieved by a modal analysis. As a result, the natural eigenfrequencies were obtained, and the mode shape of the corresponding mode (resonance) was elaborated.
- In the second part, the overall Lorentz forces generated by the EMAT inside the aluminum core were evaluated by means of a 2D simulation. The excitation frequency for this simulation was chosen based on the result of the modal analysis. The simulated Lorentz forces served as the mechanical excitation for the last simulation study.
- In the third simulation study, we evaluated the actual interaction of the EMAT with the stationary ring. In this simulation, a specific resonance of the ring was excited, and the positions for the EMAT and the receiver (accelerometer), used in the experimental study, were determined.

### 3.1. Modal Analysis of the Stationary Ring

**Figure 4:**
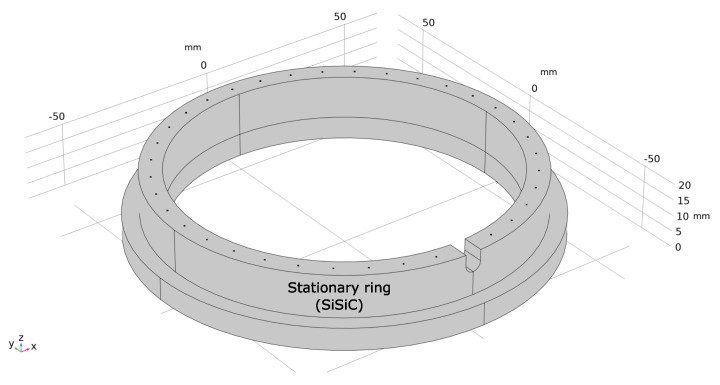
Model of the stationary ring for evaluating the eigenfrequencies and the corresponding mode shape.

To find the natural eigenfrequencies of the stationary ring, a modal analysis was carried out in COMSOL Multiphysics^®^ using the Structural Mechanics Module. In the first step, the stationary ring was modeled. The model of the ring is depicted in Figure 4.

For the modal analysis, the mechanical quantities (Young’s modulus, density, and Poisson’s ratio) of SiSiC were required. Different mechanical seals from different manufacturers provide similar values for Young’s modulus, density, and Poisson’s ratio. The parameters used in this study for the SiSiC ring are given in Table 1. These are the average values of the databases from the literature [43,44,45]. The values for aluminum were not required. They were needed for a further simulation in Section 3.3.

**Figure 5:**
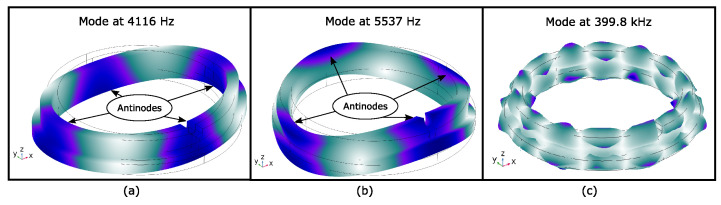
Selected modes of the stationary ring for graphical illustration. (**a**) Mode at 4116 Hz. The ring has 4 antinodes, and the preferred direction of oscillation is in the xy-plane (**b**) Mode at 5537 Hz. The ring also has 4 antinodes, but the preferred direction of oscillation is in the z-direction. (**c**) Mode at 399.8 kHz. The preferred oscillation direction is no longer unique, and the size of an antinode is smaller than that in (**a**,**b**).

Given the ring model and the mechanical properties of SiSiC, modal analysis was performed. The first two modes of the stationary ring were found at 4116 Hz and 5537 Hz. Note that this analysis did not give accurate results regarding the actual displacement of the ring. However, the modal analysis allowed the visualization of the mode shape for the computed eigenfrequency. The mode shapes of the first two modes are pictured in Figure 5a,b. The deformation is magnified by a factor of 50,000.

As can be seen, the first mode depicted in Figure 5a shows four defined antinodes. This mode preferably oscillates in the xy-plane. The ring at this eigenfrequency performs a radial movement. The second mode in Figure 5b shows also four antinodes. However, this mode shape shows a preferred direction of oscillation in the z-direction. For comparison, the mode depicted in Figure 5c shows the oscillation if the ring is excited by a transmitter, e.g., a 5 mm piezoelectric disc, which has a radial resonant frequency of around 400 kHz[31]. At this frequency, the ring no longer shows a preferred direction of vibration compared with the modes at (a) and (b). Understanding how the ring oscillates is already difficult at this frequency, and the size of the antinodes is also smaller than for the modes at (a) and (b). In addition, the distance between two adjacent antinodes has decreased. This makes a defined positioning of a receiver quite difficult. Therefore, choosing an excitation frequency smaller than 10 kHz allows a good understanding of the ring oscillation pattern and provides defined spatial antinodes on which a receiver can be mounted.

Given the result of the modal analysis, the next step was the simulation of the EMAT, especially the Lorentz force generation within the aluminum core of the PCB.

### 3.2. Lorentz Force Simulation

For Lorentz force generation, a static magnetic field and eddy currents are necessary. The resulting forces were computed according to Equation (1). The simulation was also performed in COMSOL Multiphysics^®^, using the Magnetic Fields and Solid Mechanics modules.

**Figure 6:**
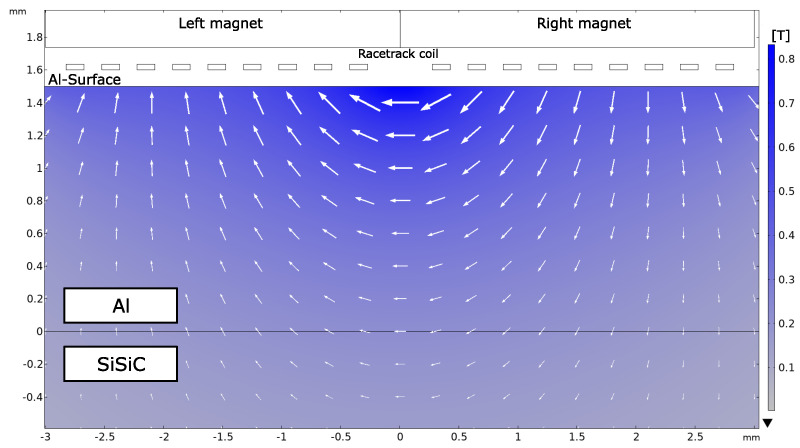
Simulated static magnetic flux density generated by a SmCo block magnet on top of the racetrack coil. Under the center of the right and left magnet, the magnetic flux density has a normal direction with respect to the aluminum surface. Beneath the center of the racetrack coil, the direction of the magnetic flux density is parallel to the surface and has the strongest magnitude.

The static magnetic field is provided by the permanent magnet block placed on top of the racetrack coil. Figure 6 depicts the simulated magnetic flux density distribution inside the 1.5 mm thick aluminum core. The right magnet corresponds to the north pole and the left magnet to the south pole of the magnet block. The remanent flux density of the SmCo magnet is about 1.08 T. The field lines of the magnetic flux density enter the aluminum layer under the right magnet and leave the aluminum under the left magnet. Directly under the magnets the direction of the magnetic flux density is almost normal to the aluminum surface. Beneath the center of the racetrack coil, the direction of the flux density changes and is parallel to the surface. In this area, the magnetic flux density shows the strongest magnitude of about 0.8 T. With increasing depth from the surface toward the Al-SiSiC interface, the magnetic flux density decreases. At the interface, the magnitude of the flux density decreases to 0.2 T.

**Figure 7:**
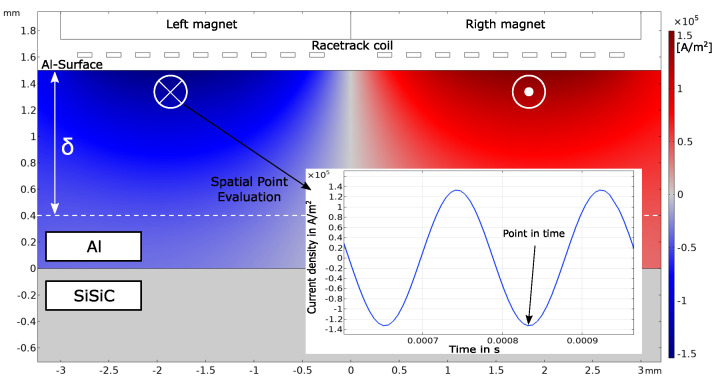
Simulation of the induced eddy current density inside the aluminum core of the EMAT. Due to the opposing direction of the driving current through the coil, the eddy currents point in different directions under the left and right part of the coil. Beneath the center of the racetrack coil, the eddy currents cancel each other out. The mandatory amount of eddy current density for an excitation frequency of 5537 Hz is within the calculated penetration depth δ according to Equation (2). The induced eddy current density follows the sinusoidal excitation current through the racetrack coil over time. As SiSiC is considered an electrical insulator, no eddy currents are induced within the SiSiC.

The second required part for the Lorentz force generation is the eddy currents. They are induced inside the aluminum core due to a sinusoidal current through the racetrack coil. The sinusoidal driving current has an amplitude of 0.5 A and a frequency of 5537 Hz. This amplitude was later used for the experiments described in Section 4, and the excitation frequency was chosen based on the result of the modal analysis. The simulation result of the induced eddy currents is shown in Figure 7. The eddy current density was evaluated at the highlighted point in time. As can be seen, the eddy current density follows the sinusoidal excitation current through the racetrack coil over time. Furthermore, it can be observed that the left part of the racetrack coil induces eddy currents pointing into the plane (blue); on the right side, the currents point out of the plane (red) because the driving current flows in the opposite direction through the coil. Below the center of the coil, the eddy currents cancel each other out. The highest magnitudes of the eddy current density can be observed directly beneath the left and right part of the coil. The magnitude of the current density decreases with increasing depth toward the Al–SiSiC interface. For an excitation frequency of f=5537 Hz, the penetration depth was calculated according to Equation (2), where $\mu_{0}=4\pi e^{-7}\;\frac{H}{m}$ is the permeability in vacuum, and $\sigma_{Al}=37e^{6}\;\frac{S}{m}$ is the electrical conductivity of aluminum. The penetration depth δ is highlighted in Figure 7. The mandatory amount of the eddy currents flow within the penetration depth. The SiSiC is an electrical insulator with electrical conductivity $\sigma_{SiSiC}=0$. Therefore, no eddy currents are induced in the SiSiC ring.

(2) $$\delta ∼\frac{1}{\sqrt{\pi \mu_{0}\sigma_{Al}f}}∼1.1\;mm$$

**Figure 8:**
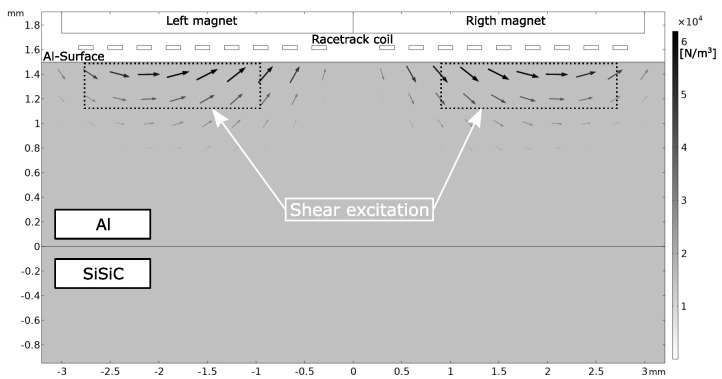
Simulated Lorentz forces based on Equation (1). Directly below the left and right magnet, Lorentz forces parallel to the aluminum surface are generated with the highest magnitude and the same direction. Beneath the center of the racetrack coil, the forces point in opposite directions and are normal to the surface. The forces penetrate about 0.5 mm deep into the aluminum. Below the middle of the racetrack coil, the forces vanish as no eddy currents are generated in this area.

With the simulations of the static magnet field and the eddy current density shown in Figure 6 and Figure 7, respectiely, the Lorentz forces could be simulated. The simulation of the Lorentz forces is depicted in Figure 8. The computation of the forces followed Equation (1). The direction and magnitude result from the cross-product of the eddy currents and static magnetic field. The strongest magnitude of the Lorentz forces was observed beneath the center of the right and left magnet. In this area, the forces point in the same direction and are parallel to the surface. Below the center of the coil, the forces point in different directions and are normal to the surface. However, the magnitude significantly decreases in this region due to the lack of eddy currents. Another observation is that the forces are generated only about 0.5 mm deep in the aluminum, although the eddy currents can penetrate deeper into the aluminum. This is due to the static magnetic field decreasing with increasing depth in aluminum.

The two excitation directions of the Lorentz force generate two different mechanical bulk waves. Lorentz forces parallel to the aluminum surface are referred to as shear excitation and produce shear body waves. Lorentz forces with direction normal to the aluminum surface are called normal excitation and generate pressure waves. Both waves types propagate toward the Al-SiSiC surface. The result of simulated Lorentz forces served as the mechanical excitation input for the upcoming 3D simulation.

### 3.3. Simulation-Based Resonance Excitation

**Figure 9:**
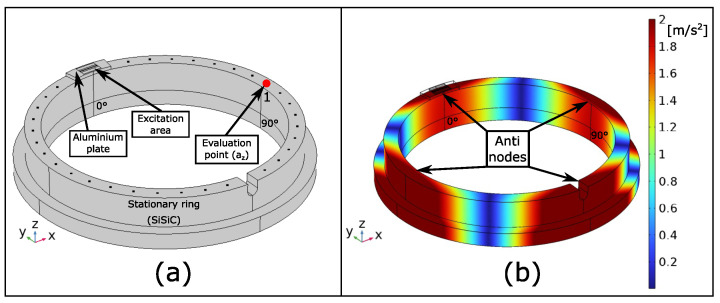
(**a**) Extended SiSiC ring model with attached aluminum plate including body loads, representing the EMAT and its excitation. (**b**) Simulated absolute acceleration in the z-direction. The simulated EMAT excitation is capable of exciting the second mode described in Section 3.1.

The goal of this simulation study was to identify the positions of the sensors (EMAT and receiver) for the upcoming experiments described in Section 4. In order to find these positions, the model of the SiSiC ring was extended. The model is depicted in Figure 9a. The ring was attached with an additional aluminum plate. This aluminum plate represented the 1.5 mm thick PCB of the EMAT. By attaching the aluminum plate, the position of the EMAT was defined at 0°. On top of the aluminum plate, two mechanical excitation areas were implemented. The mechanical excitations were body loads in the y-direction (shear excitation) and z-direction (normal excitation) according to the simulation results in Section 3.2. The study ran in the frequency domain, where the frequency of the excitation forces was set to 5537 Hz. At this frequency, the second mode, found in Section 3.1, was intended to be excited.

In order to run the simulation, the aluminum plate hX to be assigned material properties. The parameters are listed in Table 1. The values for aluminum were the default values provided by COMSOL Multiphysics^®^. The result of the simulation was the absolute value of the acceleration in fhe z-direction $|a_{z}|$. The simulation result is plotted in Figure 9b. As can be seen, the EMAT excitation manages to excite the second mode described in Section 3.1. The mode has four antinodes showing a maximum absolute acceleration of $|a_{z}|=2\frac{m}{s^{2}}$.

This mode shape now clearly allowed us to define the position of the receiver to measure the mechanical resonance in an experimental setup. The receiver was placed on one of the highlighted antinodes in Figure 9b. For the upcoming experimental setup, the receiver was attached on the antinode found at the 90° position. The EMAT position was defined at 0°.

## 4. Experimental Study

**Figure 10:**
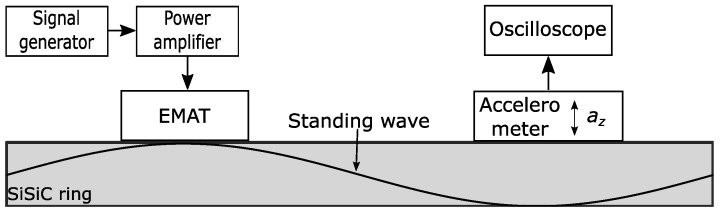
Block diagram of the measurement setup. The signal generator output provided a sinusoidal signal. The signal was amplified and fed toward the EMAT. The EMAT continuously drove mechanical waves into the ring, generating a mechanical oscillation inside the ring. An accelerometer measured the acceleration in the z-direction.

To confirm the simulation result described in the last section, an experimental study was carried out. The goal of this experiment was to meterologically demonstrate that the EMAT is capable of exciting the first two modes described in Section 3.1. Therefore, a frequency sweep was carried out between 3 kHz and 7 kHz. Figure 10 depicts the overall setup by means of a block diagram. In order to continuously drive the EMAT, further control hardware was necessary. A signal generator provided a sinusoidal signal. A standard signal generator, however, was not capable of driving the EMAT. Therefore, the output of the signal generator was amplified with a power amplifier (PA04) from Apex Microtechnology (Tucson, AZ 85741, USA). The amplifier could provide currents up to 20 A. The amplitude of the current was set to 0.5 A. This current continuously drove the EMAT attached on the ring, and a mechanical standing wave was generated inside the SiSiC ring. A MEMS-based accelerometer (ADXL 1005) was mounted on the ring as well and used to measure the oscillation in the z-direction. The accelerometer had a cut off frequency of 23 kHz, and the output was fed into an oscilloscope for data acquisition.

**Figure 11:**
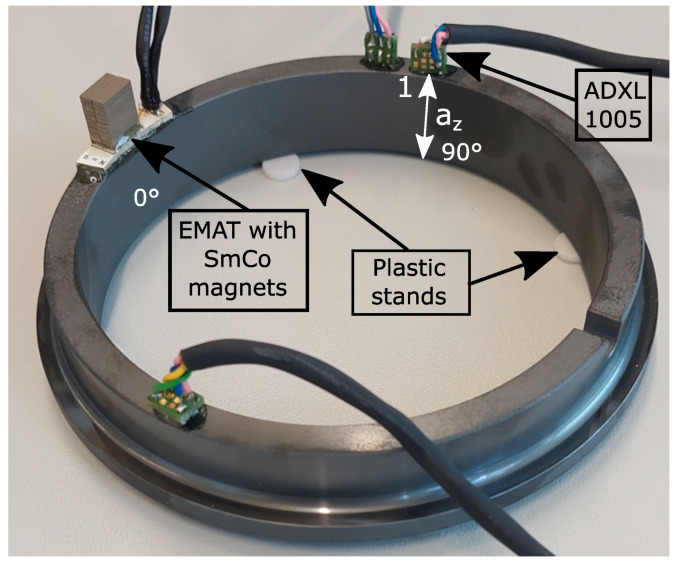
Experimental setup for the metrological evaluation of the mode excitation. The EMAT, with its SmCo magnets, was glued on top of the ring at the 0° spot and generated a continuous oscillation within the SiSiC ring. The ADXL 1005 was sensitive in the z-direction and measured the acceleration at the 90° position. The remaining two accelerometers were not used in this experiment.

The actual measurement setup used for the experiments is depicted in Figure 11. As can be seen, the EMAT nicely fit on the circular ring. It was glued at the defined 0° position on top of the ring using an epoxy adhesive (UHU Plus endfest 300). By gluing the EMAT on top of the ring, the ring acted as heat sink, and the EMAT could be continuously driven without overheating. The ADXL 1005 was also attached on the ring using the same epoxy adhesive. The accelerometer was positioned at the 90° spot on the ring. The accelerometer was soldered on a PCB, which was designed to also fulfil the requirements of the limited mounting room. The accelerometer measured the acceleration in the z-direction. The remaining two accelerometers depicted in Figure 11 were not used in this experiment. Four plastic stands lifted the ring to enable a free oscillation of the ring. They were placed at the nonoscillating nodes depicted in Figure 9b.

The excitation frequency of the sinusoidal current through the coil was varied between 3 kHz and 7 kHz, and the amplitude of the current through the coil was fixed at 0.5 A. The acceleration was measured at the 90° position.

Furthermore, a simulation using the model in Section 3.3 was performed to compare the actual measurement result with the simulation result. Here, the frequency of the body loads was also varied between 3 kHz and 7 kHz, and the magnitude of the acceleration in the z-direction was evaluated at the “Evaluation point 1”, shown in Figure 9a. This evaluation point corresponds to the 90° position of the accelerometer.

**Figure 12:**
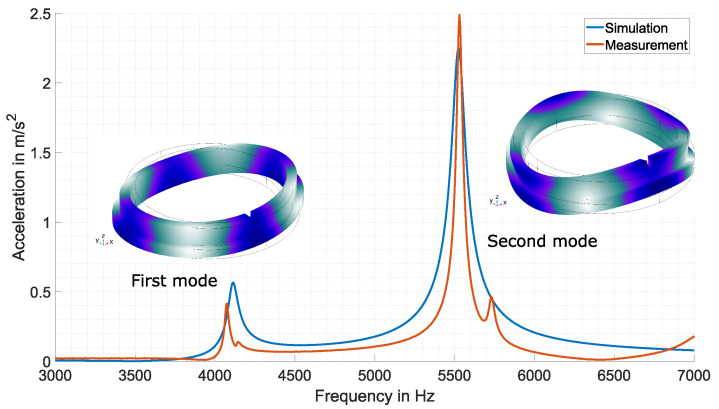
Measured frequency response and comparative simulation of the SiSiC ring. The measurement result proves the excitation of the first two modes by the EMAT. The simulation outcome confirms this observation, as the simulation shows overall agreement with the measurement. For a better illustration, the mode shape for each mode is plotted next to the corresponding peak.

Figure 12 depicts the result of the measurement and simulation. The magnitude of the measured acceleration shows two dominant maxima. The first maxima lies at $f_{1}=4075\;Hz$, and the second maxima is located at $f_{2}=5530\;Hz$. Comparing these two frequencies with the evaluated eigenfrequencies in Figure 5, the first maximum corresponds to the first mode (radial resonance), and the second maximum represents the second mode (resonance in the z-direction). The magnitude of the acceleration confirms the mode shapes of the ring, as the first mode at $f_{1}$ preferably moves in the radial direction and the second mode at $f_{2}$ shows a preferred oscillation in the z-direction. This result clearly shows that the proposed EMAT is able to excite the resonances of the ring in a desirable way.

The simulation confirms this statement. When comparing the measurement result with the simulation result, an overall agreement can be observed. The simulation also shows two distinct maxima, and the positions of the simulated maxima are in the vicinity of the measured maxima. Especially, the frequency of the second mode fits the measurement result well. The first maxima shows a slight difference with respect to the frequency. The simulation shows a maximum at 4114 Hz. Regarding the magnitudes, the maximum of the simulated accelerations differ from that of the measurement. At the first mode, the simulation shows a higher magnitude than the measurement. At the second mode, the magnitude of the simulation is lower. A slight model adjustment in terms of geometry and material parameters can be performed to correct the differences with respect to the magnitude. However, the simulation at this point was carried out to verify the eigenfrequencies between simulation and measurement result. One more difference can be observed. There are two small peaks right next to each mode in the measurement result. The origin of these peaks must be due to an interaction between the ring and the attached accelerometer, because peaks were not observed in the simulation. However, the magnitude of the acceleration of these peaks is small compared with the actual magnitude of the mode.

## 5. Discussion and Outlook

This paper presented a new EMAT capable of exciting the fundamental mechanical resonances of a stationary ring used in mechanical seals. The results show that the first two resonances occur at the eigenfrequencies of 4074 Hz and 5530 Hz. The EMAT was designed to meet the requirements of exciting the eigenfrequencies and the requirements of the maximum transmitter size due to the mounting area on the ring. Furthermore, in this study, the EMAT was continuously driven and not pulsed, as in related studies.

The result in Figure 12 clearly depicts the excitation of the first and second modes of the ring by the EMAT. The additional simulation study confirms this finding. The simulation study also illustrates the mode shapes of the first two modes of the ring. The visualization clearly shows that the first two modes each have four antinodes. The found antinodes allow a defined determination of the accelerometer position for measuring the preferred oscillation of the ring. An overall advantage of the proposed EMAT design is that it can easily be adjusted to other ring diameters. Other ring diameters have different eigenfrequencies; however, the EMAT itself does not have its own mechanical resonance. Hence, the excitation frequency can be accordingly adapted.

A further finding is the interaction between the EMAT and the ring. The stationary ring is made of SiSiC, which is a nonconductive and nonferromagnetic material. Normally, an EMAT cannot generate mechanical waves in such a material. However, the novel EMAT design also allows the excitation of mechanical waves in this material by using a PCB consisting of an aluminum core. However, we made a compromise here. We developed a transmitter that meets the requirements for excitation frequency and limited sensor size; however, we lost one property of EMATs, which is the noncontact generation of mechanical waves inside the specimen. Similar to a piezoelectric transducer, the EMAT is also attached to the ring with an adhesive material. In this regard, further research is needed on the interaction between the EMAT, adhesive, and ring. In the simulation, the adhesive layer was skipped, and the results agreed well with the measurements. However, as the excitation frequency increases, the wavelength of the mechanical waves approaches the thickness of the adhesive layer. Then, the coupling between the aluminum plate and the ring will be significantly affected.

Another issue that is part of future research is the influence of temperature drift. With respect to the EMAT, two considerations must be made. First, the permanent magnet must be considered. As the temperature increases, the remanent flux density of the permanent magnet decreases until the Curie temperature is reached. At this point, the permanent magnet becomes demagnetized. A first solution is to use SmCo magnets, as presented in this paper. SmCo magnets have a Curie temperature of about 300 °C. The usage of SmCo magnets already increases the temperature range compared with that of neodymium magnets with a Curie temperature of about 120 °C. A second consideration for the EMAT is the internal resistance of the coil. As the temperature increases, the resistance increases, resulting in less current flow through the coil. A control loop is required here to always drive the same amount of current through the coil.

Another future study topic is the actual use of the mechanical resonances excited by the EMAT. The excited resonances may provide a new fundamental basis for observing the interaction of the stationary ring with the rotating ring. However, it is first necessary to investigate which of the excited mechanical resonances are sensitive to possible interactions with the rotating ring and how the resonances are affected when the ring is installed in a test rig or even in a practical application.

## Figures and Tables

**Table 1:** Mechanical parameters used in the simulation studies.

Material	Young’s Modulus	Density	Poisson’s Ratio
Silicon-infiltrated silicon carbide (SiSiC)	365 GPa	$3128\;\frac{kg}{m^{3}}$	0.17
Aluminum (Al)	70 GPa	$2700\;\frac{kg}{m^{3}}$	0.33

## Data Availability

Not applicable.
